# From efficacy to effectiveness: a comprehensive framework for monitoring, evaluating and optimizing seasonal malaria chemoprevention programmes

**DOI:** 10.1186/s12936-024-04860-y

**Published:** 2024-02-02

**Authors:** Monica Anna de Cola, Elisabeth G. Chestnutt, Sol Richardson, Matthieu Baudry, Chuks Nnaji, Taiwo Ibinaiye, Azoukalné Moukénet, Kunle Rotimi, Benoît Sawadogo, Joshua Okafor, Cheick Saïd Compaoré, Chibuzo Oguoma, Christian Rassi, Arantxa Roca-Feltrer

**Affiliations:** 1 Malaria Consortium, 244-254 Cambridge Heath Rd, Cambridge Heath, London, E2 9DA UK; 2 Vanke School of Public Health, Tsinghua University, Beijing, China; 3 The Woodland Trust, London, UK; 4 Malaria Consortium, Abuja, Nigeria; 5 Cheikh Anta Diop University, Dakar, Sénégal; 6 Malaria Consortium, Ouagadougou, Burkina Faso; 7 PATH, Maputo, Mozambique

**Keywords:** Monitoring and evaluation, Process evaluation, Impact evaluation, Decision-making, Malaria interventions, Program optimization

## Abstract

**Background:**

Seasonal Malaria Chemoprevention (SMC) is a highly effective intervention for preventing malaria, particularly in areas with highly seasonal transmission. Monitoring and evaluating (M&E) SMC programmes are complex due to the scale, time-sensitive delivery of the programme, and influence of external factors. This paper describes the process followed to develop a comprehensive M&E framework tailored specifically for the SMC context.

**Methods:**

The Framework was developed through a literature and programme review, and stakeholder dialogues across three implementing countries—Burkina Faso, Chad, and Nigeria. Expert consultation further refined the Framework through an iterative approach drawing upon data collected through the three sources. The Framework was designed using the Logical Framework Approach incorporating external factors and intentionally aligned with global malaria M&E standards.

**Results:**

An overall aim and seven programme objectives were developed measured by 70 indicators. The indicators also capture the causal links between the implementation and results of the programme. The Framework leverages the use of current data sources and existing mechanisms, ensuring efficient data use without requiring a significant increase in resources for overall programme optimization. It also promotes the use of data triangulation, and stratification for a more nuanced understanding of factors affecting programme performance and timely data informed decision-making.

**Conclusions:**

The SMC M&E Framework presented here provides a standardized approach for programme implementers to enhance decision-making for optimal programme performance. This is an essential tool as the scope of SMC programmes expands to new geographies and target age groups.

**Supplementary Information:**

The online version contains supplementary material available at 10.1186/s12936-024-04860-y.

## Background

Since 2012, the World Health Organization (WHO) has recommended the delivery of seasonal malaria chemoprevention (SMC) to prevent malaria in areas with highly seasonal transmission [1]. SMC involves administering sulfadoxine-pyrimethamine and amodiaquine (SPAQ) at monthly intervals during the transmission season [2]. Efficacy studies have shown SMC is a highly effective method to prevent malaria, averting over 75% of uncomplicated and severe cases in children under five [3]. However, when delivered in real-world settings the results have varied considerably, demonstrating a gap between the results observed in controlled environments and the impact achieved on the ground [4–6]. The cause of the gap between efficacy and effectiveness remains unclear.

During the implementation of SMC programmes monitoring and evaluation (M&E) is used to identify challenges by tracking coverage and impact indicators. However, there are no universally agreed indicators or objectives SMC programmes are required to follow and many programmes carry out M&E on an ad hoc basis. The absence of standardization hinders spatial and temporal comparison. Moreover, several other factors rarely included in evaluations can influence the impact of an SMC programme, such as the quality of implementation and contextual or environmental features. In addition, evaluating malaria programmes is particularly challenging as they are often implemented without baseline data collection or comparator areas, which results in a lack of an appropriate counterfactual [7]. Monitoring these additional aspects may help to explain the efficacy-effectiveness gap and provide valuable insights for optimizing the impact of SMC.

A framework was developed to provide SMC implementers with a comprehensive and standardized approach for monitoring and evaluating SMC programmes. The framework supports programmes to optimize performance and improve effective decision-making and priority setting. In addition, providing a standardized approach allows for comparison of results between countries and contexts. This is particularly important given the recent update of the WHO’s SMC guidelines, which will provide more flexibility to potentially expand the target populations [8]. Since its creation, the value of this Framework has been recognized by other organizations and has informed the development of the SMC Alliance’s Performance Framework in the Seasonal Malaria Chemoprevention Monitoring & Evaluation toolkit [9], and the World Health Organization’s SMC Field Guide [2]. This paper outlines the methodology followed to develop the SMC M&E Framework.

## Methods

### Framework development process

**Fig. 1:**
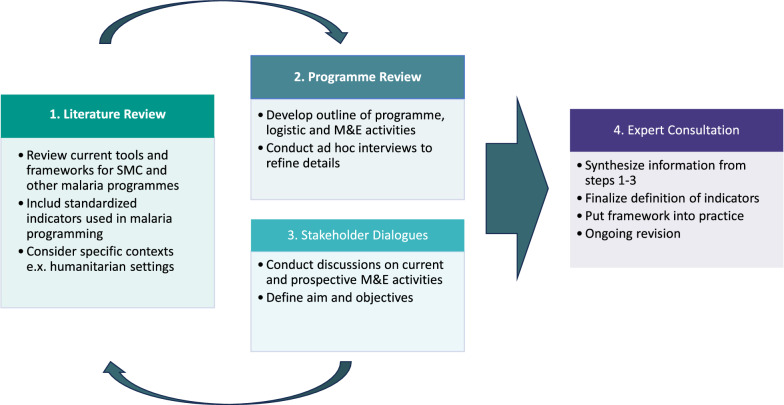
Flowchart of framework development process

The framework development process is outlined in Fig. 1. The data gathering process involved a literature review, programme review and stakeholder dialogues. This information was then reviewed by a working group of experts in the subject area. The members of the working group included individuals with advanced qualifications or more than five years of practical experience in the following areas: malaria, SMC, M&E, programme implementation, research, logistics, mass drug distribution campaigns, social and behavioural change communication and clinical case management.

### Literature review

A literature review was conducted to identify existing frameworks used for monitoring and evaluating public health programmes. A search of the PubMed database was conducted using the following terms: “monitoring and evaluation frameworks” AND “malaria control programmes.” To supplement the white literature and ensure representation of programmatic and implementation perspectives, a Google search was carried out to identify grey literature using the following terms: “monitoring and evaluation frameworks”, “M&E frameworks”, “monitoring public health interventions”, and “monitoring malaria control programmes”. Grey literature was also identified by searching websites of organizations with recognized expertise implementing and researching public health interventions including: The University of Oxford, Save The Children, Measure Evaluation, United States Agency for International Development, the United Nations Office for Disaster Risk Reduction, and the WHO. The research team screened the results for relevance, with a particular focus on frameworks developed for malaria programmes.

### Programme review

In 2019, a programme review was conducted in three countries—Burkina Faso, Chad and Nigeria—to understand the processes and activities carried out during an SMC programme. These countries were selected as they had established SMC programmes that had been implemented for six or more years and represented diverse contexts to ensure that the framework accounted for a variety of situations.

During the field visits the research team observed and documented the microplanning, procurement and delivery and data collection and collation processes. This included the data collection tools, data flow, personnel roles and responsibilities, and the established timelines for data reporting. As part of the programme review process, the team discussed their observations with the programme teams and clarified any processes and activities to enhance the teams understanding of contextual factors affecting each programme and ensure accurate documentation. The documentation from the field visits was synthesized for the working group to review.

### Stakeholder dialogues

In November 2019, the team conducted a series of stakeholder dialogues with representatives from the three study countries. The stakeholder dialogues consisted of three structured conversations to gather input, feedback, and perspectives on the current and prospective M&E activities of the programme. During the stakeholder dialogues, qualitative information was gathered to guide the decision-making process for the structure and design of the SMC M&E Framework. This included defining the overall aim and objectives.

### Expert consultation

The synthesized findings of the literature and programme review and stakeholder dialogues were reviewed by the expert working group. To ensure the working group covered breadth and depth of knowledge, core members included M&E, operations, and logistics specialists. Additional participants were invited on an ad hoc basis when an appropriate topic was discussed. Ad hoc members included: researchers, technical experts, and country- and regional- level decision-makers. Malaria Consortium is a leading implementer of SMC therefore the majority of working group participants were from this organization.

Between October 2019 and November 2020, the group met fortnightly to discuss existing knowledge, best practices, and gaps to inform the creation of the Framework. Meetings were held through an online platform due to the COVID-19 pandemic. The sessions were chaired by an M&E expert who guided the review and discussion of the evidence. The development of the Framework followed an iterative approach, which ensured each source of evidence contributed to the design and refinement of the Framework. After each session, the M&E expert updated the Framework and shared the revised version with the group for discussion at the next meeting. This approach also ensured the Framework was built through collective expert knowledge and consensus.

## Results

### Literature review

The search of the PubMed database identified 44 articles covering M&E frameworks, methodologies, and best practices for public health programmes. In addition, a range of documents, reports, M&E templates, and guidelines were identified, offering valuable insights into M&E frameworks across different contexts, including different levels of risk and security.

Three peer-reviewed articles were most relevant for this study, two of which were produced by the RBM Partnership to End Malaria’s Surveillance, Monitoring and Evaluation Working Group (SME WG) [10–12]. The SME WG’s evaluations of malaria control efforts in high-burden Sub-Saharan African countries [10] and their most recent work in moderate- and low- transmission settings [12] heavily informed the overall structure of the Framework and the inclusion of external factors. Several of the indicators in these works were also included in the final which ensured the final version of the Framework was aligned with global standards for malaria M&E programming.

### Programme review

The programme review provided information on the operational processes and the tools and methods used to monitor and evaluate implementation of SMC programmes.

### Delivery method

During the field visits, it was observed that SMC was typically delivered door-to-door by community distributors, who administered the preventative medicines to the target population. Community distributors used specific terminology to refer to different parts of the delivery. The protective period provided by a full course of SPAQ was referred to as a “cycle”. Each cycle was delivered over four consecutive days and involved the administration of a 3 day regimen of SPAQ, referred to as a “course”. In most settings, an annual “round” of SMC included four cycles, each delivered 1 month apart. However, in some areas the number of cycles in a round was shortened or extended, depending on the duration of the high-transmission season. In all cases the number of cycles in a round was between three and five. The term “SMC campaign” was used to refer to all the activities required to implement SMC, including those occurring before and after the distribution of the medicines. These activities included planning and enumeration, procurement and supply management, community and stakeholder engagement, training, case management and pharmacovigilance, supervision, and M&E.

### Data collection tools

Several data collection tools were used routinely to document SMC activities throughout the pre-implementation planning, implementation, and post-implementation. During the pre-implementation, data collection tools were used in both macro- and micro-planning to quantify the resources required for procurement, create detailed activity plans and estimate the resources required for distributing at community level. The data collection tools were completed once per round, prior to the commencement of the first cycle and the data was entered into microplanning database to track the inputs required for the programme including commodities, supplies, and human resources.

During the implementation phase, community distributors used tally sheets to record the number of children treated, logistics accounting and pharmacovigilance data. Following the completion of each cycle the tally sheets were summarized in end-of-cycle report forms which reported the number of children reached during the cycle at the lowest health facility catchment level.

In addition, surveys were conducted after each SMC cycle, up to the penultimate cycle. These surveys provided real-time information for in-process monitoring. For example, the end-of-cycle household surveys used a lot quality assurance sampling methodology to monitor SMC coverage and quality. This enabled implementing teams to identify any issues, such as areas of low coverage, and make adjustments before the start of the next cycle. After the final SMC cycle end-of-round surveys were completed instead. These representative household-level surveys were comprehensive and collected data to estimate coverage during each cycle of the round and to evaluate the quality of delivery in the final cycle. These surveys also collected household- and individual-level data, such as sociodemographic characteristics and health-seeking behaviour to provide contextual information for the team during the analysis of the routine programme data.

### Stakeholder dialogues

Stakeholder dialogues were conducted with 22 individuals with diverse knowledge and experience to ensure the Framework aligned with a variety of programme priorities and objectives. The participants included five M&E and epidemiology experts, six operations and security specialists, seven programme implementers, one surveillance specialist, one research expert, one learning specialist and one case management expert. The participants also represented diverse geographical experience with seven global-level experts and fifteen national-level experts from four countries—Burkina Faso, Chad, Mozambique and Nigeria.

The stakeholder dialogues identified a lack of standardized aims and objectives for SMC programmes, which served as a crucial starting point for the M&E framework. The information from stakeholders also helped to identify key indicators necessary for measuring the programme’s success and gaps in the current M&E data collection. The data collection gaps varied in different contexts which emphasized the need for a flexible framework that could be adapted to different implementation strategies.

### Conceptual design

**Fig. 2:**
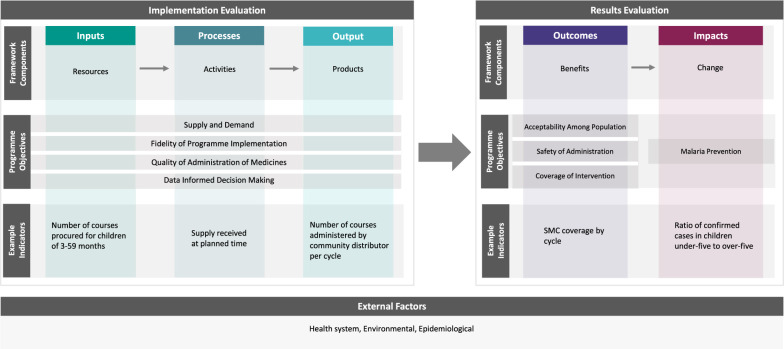
SMC M&E Framework Conceptual Design: from inputs to impact

The SMC M&E Framework is structured using the Logical Framework Approach, where each component—implementation and results—is causally linked (Fig. 2) [13]. This is a common approach used in modern M&E frameworks due to its structured and systematic nature [14, 15]. The Framework considers the underlying relationship between the resources, activities, products, desired benefits, and expected changes, by capturing the implementation, outcomes, and impacts in succession. There are two evaluation components: implementation evaluation and results evaluation. The implementation evaluation—consisting of inputs, processes and outputs—measures the degree to which a programme is implemented as intended and assesses the quality of the implementation. The level of achievement of the programme, measured through the implementation evaluation, directly impacts the level of achievement in the results evaluations. The results evaluation consists of the outcomes, which assesses the results of the programme and how the results affect the target population, and the impacts, which measures the longer-term effects of the intervention and is defined in relation to the overall programme aim.

### Defining aims and objectives

Having identified the need to define programme aims and objectives during the stakeholder dialogues, the working group agreed upon one overarching aim and seven objectives which are outlined in Table 1. The aim reflects the purpose of SMC programmes and each of the objectives represents an essential component that must be completed to successfully achieve the aim of the programme. For instance, even if the programme is executed with utmost fidelity, its impact and coverage will remain low if community acceptance is low. To achieve optimal programme outcomes, it is imperative to consistently track all the objectives from inception to conclusion to identify areas that have been successful or require improvement.

**Table 1:** SMC M&E Framework aim and objectives

	Description	Short name
Goal	To safely^a^ prevent malaria cases^b^ in eligible children^c^living in areas targeted^d^ by the seasonal malaria chemoprevention (SMC) programme^e^ within the intended period of protection^f^	Malaria prevention
Objective 1	Maximize the number of eligible children reached and receiving the correct dose of SPAQ in targeted areas	Coverage
Objective 2	Ensure complete reporting of, and minimize occurrence of, significant adverse events following drug administration, including monitoring contraindications and other reactions to treatment, to ensure safe use of sulfadoxine–pyrimethamine plus amodiaquine	Safety
Objective 3	Gather, and make effective use of, information obtained from monitoring and evaluation activities to inform decision-making in a timely manner, and drive short- and long-term programme improvements	Decision-making
Objective 4	Secure the highest-possible degree of acceptability among caregivers of eligible children	Acceptability
Objective 5	Ensure the highest-possible quality of programme delivery in all aspects	Quality
Objective 6	Achieve the highest-possible fidelity of programme delivery	Fidelity
Objective 7	Ensure provision of appropriate inputs to meet programme demands in relation to the place, time and person	Supply and demand

^a^Without severe adverse events resulting from SMC administration

^b^Severe and uncomplicated

^c^Defined as meeting the current eligibility criteria for eligibility for SMC as recommended by the WHO, including being within the specified age range (3–59 months), absence of allergy, confirmed malaria or other acute illness, and other criteria

^d^Encompassing the geographic area or administrative unit(s) designated for coverage by the campaign, irrespective of actual geographic coverage

^e^In this instance, defined as intermittent prophylactic administration of sulfadoxine–pyrimethamine plus amodiaquine within a defined high-transmission season

^f^In the case of SMC with sulfadoxine–pyrimethamine plus amodiaquine, each course confers protection for 28 days; assuming monthly intervals between SMC cycles are maintained, the intended period of protection therefore includes the time from administration of the first course of SMC until 1 month after administration of the last monthly cycle in an annual SMC round

### Defining key indicators

The working group defined key indicators for each of the objectives and grouped these by the implementation and results components of M&E, as provided in Additional file 1. The 70 indicators, derived from 17 sources, enable M&E teams to track progress and identify if an objective has been achieved. For example, an input indicator for achieving the supply and demand objective would be the number of treatment courses procured. Each indicator has a clearly defined target, to provide a benchmark for monitoring progress and defined parameters, such as aggregation level (e.g., geographic area), stratification type (e.g., age), and reporting frequency. Each indicator also includes any available baseline data and the data source, for tracking progress to improve standardization. Furthermore, during the development of the Framework the working group identified a need to record the assumptions associated with each indicator. The assumptions outline the conditions under which the programme is expected to operate successfully. Any deviation from the assumed conditions can affect programme performance and tracking and identification of these factors can help to interpret abnormal results or outcomes.

In addition to the standardized elements the Framework is designed to allow programmes to include additional data that is not routinely collected but appropriate for the context. Data sources may include operations trackers, budget documentation, training reports, work plans and supervision reports. The Framework can also capture information on personnel roles and responsibilities to improve accountability.

### External factors

The implementation and results of an SMC programme are influenced by the external environment. For instance, during the programme review in Burkina Faso, a health worker strike occurred, which prevented programme implementers from collecting routine programme data [16]. This impacted the process component of the campaign, and without contextual information the programme data may inaccurately suggest fewer children had received monthly SMC doses in that year. As a result, the working group identified several external factors that can influence each of the M&E components and categorized them into three groups: health system factors, environmental factors, and epidemiological factors. Health system factors include the availability of commodities and disruptions in access to health services. Environmental factors include meteorological variables influencing vector dynamics, along with natural and human-induced events impacting programme implementation. Epidemiological factors influencing programme outcomes include coverage of other malaria interventions and the baseline prevalence of malaria in targeted populations.

Including data on the external environment in the Framework provides contextual information to support the interpretation of results and outcomes of the programme. This information can serve as co-variables in adjusted analyses to account for their effects on programme impact or help to identify and address implementation issues. Overall, incorporating external factors contributes to achieving a high-quality programme implementation through a tailored approach.

### Programme evaluations

The Framework is designed to allow implementers to evaluate the programme at two levels: implementation and results.

### Implementation evaluation

The implementation evaluation component combines the required resources (inputs), operational aspects of the programmes (processes), and the services provided (outputs) to characterize the overall implementation of the SMC programme. A total of 45 indicators allows the programme team to identify whether the programme has met the intended outcomes and impacts as defined by the aim.

The implementation evaluation begins with tracking the input indicators for the programme. Most of the data to populate these indicators originate from microplanning and operations trackers and can be entered into the Framework in advance of the first cycle to better understand the programme’s scale.

The data for the process and output indicators are then entered during the implementation. The data sources for these indicators are typically end-of-cycle reports, supervisor checklists, training reports, inventory control cards, the work plan, and end-of-cycle surveys. Considering the time-sensitive nature of SMC, these should be entered into the Framework after each monthly cycle to ensure the data can be used to make necessary adjustments.

### Results evaluation

The results evaluation consists of indicators to assesses the programmes outcomes and impacts. The 18 outcome indicators allow implementors to determine whether the SMC programme has achieved the expected level of coverage and if the coverage is equitable. To achieve maximum impact of the SMC programme, each dose of SPAQ must be administered on time and coverage should remain high for each cycle throughout the SMC round.

Therefore, the outcome indicators measure overall coverage, coverage for each month of the administration plan, and adherence to each course. In addition, the Framework contains indicators to measure the outcomes of other defined objectives such as fidelity, quality and acceptability. Typical data sources to populate these indicators include representative and end-of-round household surveys which collect information including caregiver knowledge, the observation of SMC being administered and correct completion of record cards by caregivers.

The impact component of the results evaluation uses eight indicators to measure the longer-term effects on health outcomes such as malaria-attributable morbidity and mortality. These evaluations must be planned before SMC is implemented in a new setting and continuously monitored throughout the programme lifetime. Continuously monitoring the impact indicators can help to detect implementation challenges and any potential changes in efficacy or resistance. A variety of data sources can be used to measure, triangulate and validate impact, these include parasite prevalence and the number of confirmed malaria cases presenting at the health facility. Secondary impact indicators, such as prevalence of anemia and hospitalization rates can also be useful data sources to measure the impact of an SMC programme.

## Discussion

Understanding the factors that influence the success of SMC programmes is critical for maximizing the impact of this intervention. Achieving the efficacy observed in controlled environments when deployed in real-world settings could have a marked impact on malaria transmission. This study found that although SMC programmes have been deployed for over ten years, the absence of standardized aims, objectives and indicators has prevented the comprehensive comparison and analysis of impacts observed in different settings.

A review of research and operational literature, observations from three established SMC programmes and stakeholder dialogues with experts at national and global levels were conducted to gain insights into the complexities of delivering SMC. The results found a variety of reasons that monitoring and evaluating SMC programmes is challenging. Most notably, SMC is delivered through a large-scale community-based intervention which requires substantial resources for planning, implementation, data collection, and analysis. In addition, the timing of SMC programmes is critical for achieving optimal impact, and the short duration of each SMC cycle requires efficient data processing to support timely decision-making. A M&E framework specifically tailored for SMC is, therefore, essential to capture both the volume of data and facilitate real-time decision-making and response. In addition, a standardized set of objectives and indicators can support the optimization of the programme and increase impact.

The SMC M&E Framework provides a holistic approach for monitoring and evaluating SMC programmes across multiple components beyond coverage and efficacy. The Framework is important for several reasons. First, the implementation of this Framework will encourage harmonization of indicators and methods across various SMC implementers and ensure that the data collected are consistent and reliable. This will support programmes to identify areas for improvement. In addition, this Framework facilitates the gathering of data from different sources, allowing for triangulation and helping implementers to understand the factors affecting the impact of their programme. By collating additional data, a more complete picture of the impact of SMC programmes can be obtained, enabling more informed decision-making and policy development. Furthermore, this Framework allows for analyses at different stratification levels and allows data to be analysed by different characteristics. For example, entering the data at the lowest administrative level allows for subnational analyses and intra-country comparisons such as urban versus rural, high versus low security risk, and new versus existing SMC geographies based on the updated WHO Guidelines [8]. This enables a more nuanced understanding of the impact of SMC programmes across different population sub-groups, allowing for more locally tailored approaches to effectively serve the hardest-to-reach target populations. Finally, the widescale use of this Framework will facilitate comparisons across different years, geographies and implementing partners which can support the sharing of learning, best practice and build the SMC evidence base to guide global-level decision-making and policy.

Overall, a standardized M&E framework for SMC plays a pivotal role in addressing optimization by focusing on enhancing the effectiveness of existing mechanisms rather than introducing entirely new approaches that may require additional funding or resources. By systematically tracking and evaluating indicators from programme inception to impact, this Framework helps identify areas where improvements can be made within the current programme structure which ultimately leads to improved malaria prevention and control efforts without necessitating a significant increase in financial investment. This is important given the current funding landscape for global health programmes.

To fully realize the benefits of this M&E framework, it is crucial to ensure the data collected are used to inform decision-making. This bridges the gap between programme implementers and M&E specialists to ensure that the data are used effectively to improve programme delivery. It is also important to measure the impact of the associated decisions and changes, to evaluate effectiveness and make further adjustments. This may involve establishing additional M&E activities to track changes in outcome and process indicators or conducting more rigorous research studies to assess the impact of programme changes over time.

SMC impact is complex and dynamic, and more extensive factors must be considered depending on the data source. Further work is planned to improve understanding of these factors and identify the most effective approaches for measuring and achieving the desired impact of SMC [17]. Overall, the identification and tracking of external factors that influence programme implementation, outcomes, and impact are critical to ensure effective programme implementation and to achieve the best possible health outcomes.

In addition, future work is needed to increase understanding of how monitoring implementation can facilitate better adjustment of impact analyses. To achieve this, it is necessary to go beyond the triangulation of data and determine which factors should be accounted for in impact analyses to adjust for confounding variables. Additionally, this Framework can benefit from increased engagement with stakeholders, including malaria programme implementers at various levels. This will help ensure that the Framework is flexible to the needs of different programmes, and that data collected are relevant and useful for decision-making. Furthermore, the learnings from using the Framework in different countries and contexts will be important for updating the key influencers and challenges to SMC implementation. Finally, regular review of the Framework is in place to ensure that it grows with the programme and remains fit-for-purpose (Additional file 1).

## Limitations

Although a standardized M&E framework facilitates the process of collating and reporting data the operationalization will require planning. The optimal use of the framework requires the timely acquisition of data which can be challenging particularly if data require validation by programme decision-makers at various levels. Additionally, though not unique to this Framework, data accuracy and completeness are important to ensure the decisions informed by the data can be made with confidence. These limitations highlight the need for effective systems and processes and resourcing for high-quality data management. In addition, the flexibility of the Framework allows additional dataset to be included which may be beneficial in some contexts. However, allowing this flexibility may lead to the collection of vast amounts of data that are not useful for analyses and add complexity to the identification of influential factors. Data collection should be planned and prioritized based on the capacity to act on the findings. One way to ensure that the data collected are useful is to define the essential standard indicators that should be collected by all stakeholders. This allows for comparison of several indicators across different settings while ensuring that the data collected is relevant for measuring programme goals and objectives set by the wider stakeholder consensus.

Some limitations in the methodology should also be recognized and considered when choosing to implement the Framework. First, the development of the Framework is predominantly based on data collected from programmes implemented by Malaria Consortium. Although Malaria Consortium is the largest implementer of SMC the organization is not the only implementer. The Framework has been designed to be adaptable; however, it is recognized that now, as the programme has expanded geographically and to other populations, insights from a wider community of stakeholders would be beneficial. Finally, the process involved a literature review rather than a systematic review, which may have resulted in limited results on the breadth and depth of information considered during the framework's development. However, the purpose of the literature review was to identify current frameworks in practice and prioritize global standards set by organizations such as the RBM Partnership to End Malaria and the WHO.

## Conclusion

The SMC M&E Framework offers a standardized method for stakeholders to identify performance issues, enhance decision-making, and make programme adjustments to achieve stated objectives. The Framework allows for harmonization of indicators and methods across different contexts to facilitate comparability and to add to the evidence base of programme impact. By strategically leveraging existing resources and fine-tuning implementation strategies based on real-time data, the M&E Framework contributes to a more efficient and impactful SMC programme. The value of this Framework has already been recognized by organizations including the SMC Alliance and the WHO. As the scope of SMC programmes expands to new geographies and age groups, the utility of this Framework will increase. However, further work is needed on identifying priority indicators for adjusting impact analyses, assessing the effect of programme changes over time, and increasing engagement and consistent use by various stakeholders. Overall, the SMC M&E Framework is a valuable tool for optimizing the use of increasingly limited resources to improve public health outcomes and accelerate progress towards malaria elimination goals.

### Supplementary Information

**Additional file 1.**

## Data Availability

Data sharing is not applicable to this article as no datasets were generated or analysed during the current study.
